# Systematic Review and Pharmacological Considerations for Chloroquine and Its Analogs in the Treatment for COVID-19

**DOI:** 10.3389/fphar.2020.554172

**Published:** 2020-10-28

**Authors:** Hongwei Peng, Zhangren Chen, Yunyun Wang, Simei Ren, Tiantian Xu, Xin Lai, Jinhua Wen, Mengjun Zhao, Chuanfei Zeng, Lijuan Du, Yanmei Zhang, Li Cao, Jinfang Hu, Xiaohua Wei, Tao Hong

**Affiliations:** ^1^Department of Pharmacy, The First Affiliated Hospital of Nanchang University, Nanchang, China; ^2^Academic Affairs Office, The First Affiliated Hospital of Nanchang University, Nanchang, China; ^3^National Center for Clinical Laboratories, Beijing Hospital, National Center of Gerontology, Chinese Academy of Medical Sciences, Beijing, China; ^4^Beijing Engineering Research Center of Laboratory Medicine, Beijing Hospital, Beijing, China; ^5^Graduate School, Chinese Academy of Medical Sciences and Peking Union Medical College, Chinese Academy of Medical Sciences, Beijing, China; ^6^The First Clinical Medical College of Nanchang University, Nanchang, China; ^7^Department of Neurosurgery, The First Affiliated Hospital of Nanchang University, Nanchang, China

**Keywords:** COVID-19, chloroquine, hydroxychloroquine, pharmacological considerations, piperaquine

## Abstract

COVID-19 has been announced pandemic by WHO and over 17,000,000 people infected (Till April 21st 2020). The disease is currently under control in China, with a curative rate of 86.8%. Chloroquine (CQ) is an old anti-malarial drug with good tolerability, which had proved to be effective in previous SARS-coronavirus, which spread and disappeared between 2002-2003. In vitro studies demonstrated the efficacy of CQ in curing COVID-19. Consequently, *via* analytical PBPK modeling, a further preliminary clinical trial has proved the efficacy and safety of CQ in China., and multiple clinical trials were registered and approved to investigate the activity of other analogs of CQ against COVID-19. We have listed all the clinical trials and made a meta-analysis of published data of hydroxychloroquine (HCQ). HCQ could increase the CT improvement and adverse reactions (ADRs) significantly though there was considerable heterogeneity among current researches. Actually, CQ and its analogs have unique pharmacokinetic characteristics, which would induce severe side effects in some circumstances. We have then summarized pharmacological considerations for these drugs so as to provide to the busy clinicians to avoid potential side effects when administered CQ or its analogs to COVID-19 patients, especially in the elderly, pediatrics, and pregnancies.

## Introduction

COVID-19 (coronavirus disease 2019) has been pandemic around the world by WHO, with millions of people infected currently and causes 5–10% of patients’ deaths. COVID-19 was first reported in December 2019, and the pathogen was firstly isolated on January 7^th^, 2020 and then named SARS-CoV-2 by International Committee on Taxonomy of Viruses (ICTV), as it shares great similarity in genomics with the previous emerged SARS-CoV. Chloroquine (CQ) was firstly found to be effective *in vitro* and then showed promising activity against SARS-CoV-2 infection (2019). Basically, CQ is a classic anti-malarial drug with an immunosuppressive effect as well. Due to the long half-life, it is more prone to be accumulated *in vivo* and cause various side effects, which may hinder the treatment of COVID-19. Here, we aim to provide rational suggestions to busy clinicians to avoid potential side effects that CQ or its analogs if prescribed to treat COVID-19.

**Figure 1:**
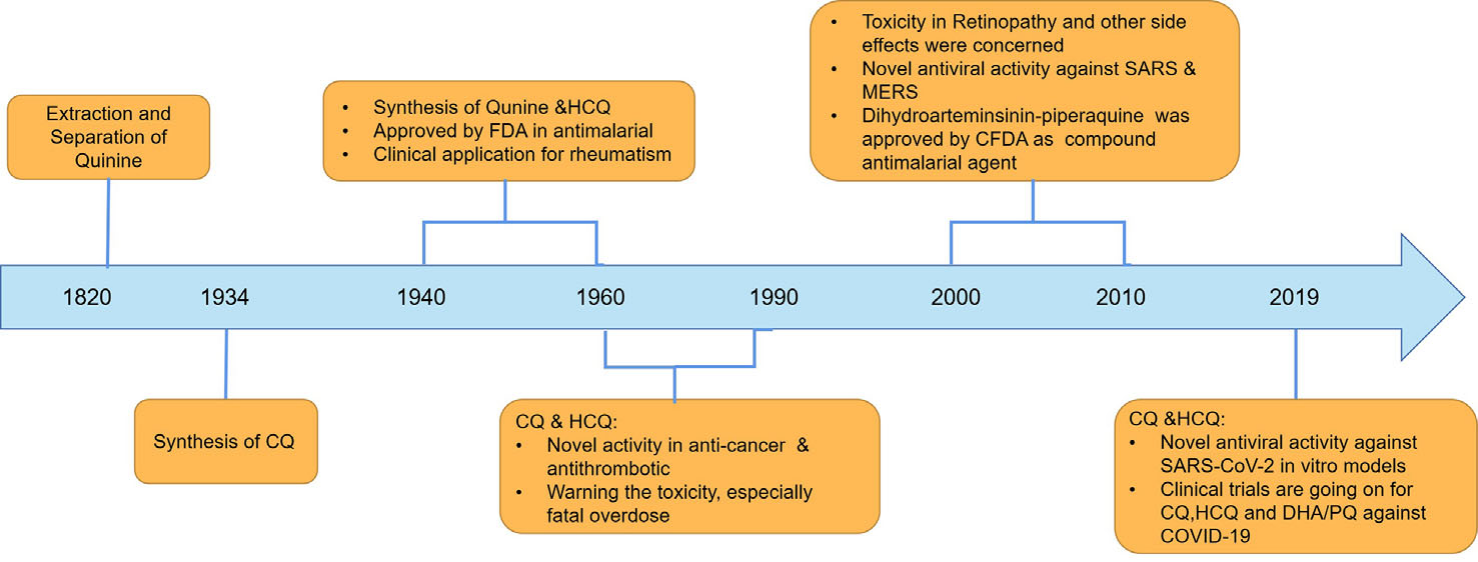
The history of CQ and its analogs in clinical application.

**Figure 2:**
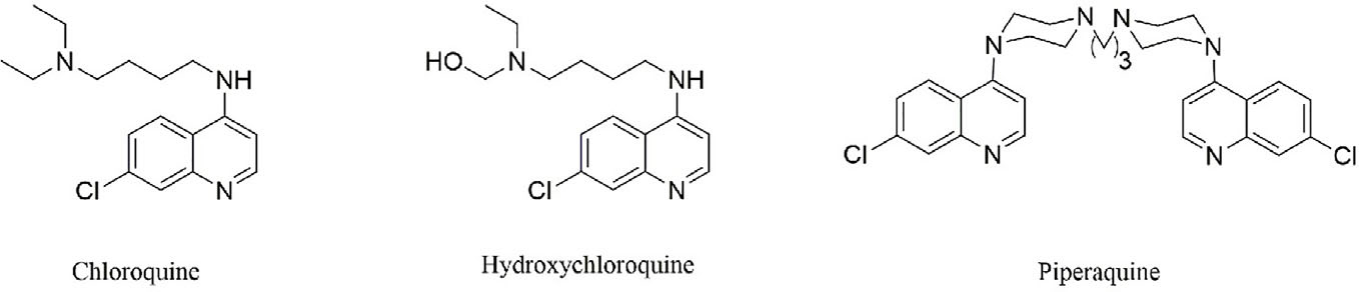
The structure of CQ and its analogs. Hydroxychloroquine differs from CQ by the presence of a hydroxyl group on one of the ethyl groups attached to nitrogen in the side chain. Piperaquine, which share the bisquinolines structure, may be more efficiently trapped in the acidic digestive vacuole and thus inhibit coronavirus entry and replication.

CQ and its analogs were used as anti-malarial agents for decades, and they were derivatives of 4-quinoline, whose origin is quinine. Actually, the anti-malarial agents of quinine were found as early as 1639, and researchers successfully extracted and separated in 1820 (**Figure 1**). Currently, CQ and hydroxychloroquine (HCQ) were used widely in anti-malarial and in the fields of SLE treatment. The differences between CQ and HCQ were the presence of a hydroxyl group in the side chain in HCQ (**Figure 2**). Though the lack of large-scale clinical trials, the efficacy and the safety were approximately identical for CQ and HCQ. It is reported that CQ was prone to be more often associated with retinopathy, especially in SLE patients, who were believed to go on a longer course of HCQ treatment (Clotilde et al., 2018). It is noteworthy that CQ and HCQ were reported to have the antiviral activity inagainst SARS [severe acute respiratory syndrome (SARS)] and MERS [Middle East respiratory syndrome (MERS)] *in vitro*, and clinical research in MERS coronavirus (MERS-CoV) confirmed the efficacy (Al-Bari, 2015; Cortegiani et al., 2020; Hoffmann et al., 2020; Wang et al., 2020). As COVID-19 is pandemic around the world, it is of great importance to explore the promising drugs and nconfirm the efficacy. As SARS-CoV-2 shares a similar structure and target of SARS-CoV, CQ’s antiviral activity has been confirmed quickly *in vitro* models by Chinese scientists, and multiple clinical trials were still going on. On March 20th 2020, FDA has approved CQ as sympathetic medication for COVID-19 patients (Bulloch, 2020; Coronavirus Task Force, 2020). However, besides their striking activity, CQ and its analogs had many side effects, which should be taken into consideration in clinical practice. The FDA cautions against use of HCQ/CQ for COVID-19 outside of the hospital setting or a clinical trial due to risk of heart rhythm problems (Bulloch, 2020). Here, we summarized the latest developments and pharmacological concerns for CQ and its analogs in order to provide rational consideration for their rational clinical application.

## The Pharmacological Mechanism of CQ Against Coronavirus Infection

**Figure 3:**
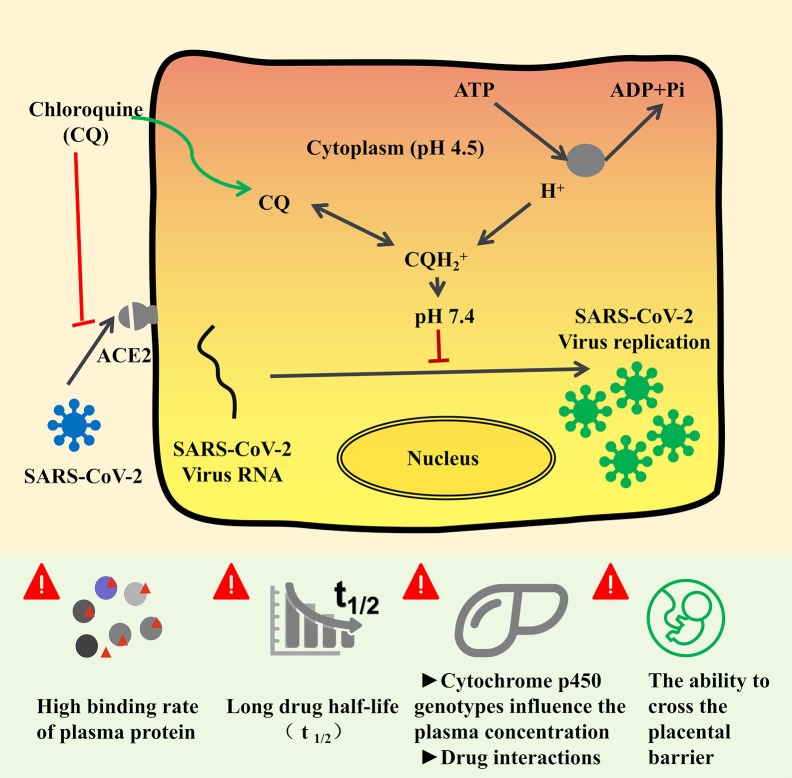
The mechanism of chloroquine and its analogs on the inhibition activity of COVID-19 (Clotilde et al., 2018; Hoffmann et al., 2020; Yao et al., 2020) and pharmacological considerations. COVID-19 infection depends on the host cell factors ACE2, which is the attachment receptor of the virus. After interfering with ACE2 and prime S protein, COVID-19 enter the host cytoplasm and initiate massive replication. Chloroquine and its analogs could inhibit the interaction between coronavirus and ACE2. On the other hand, CQ could increase the cytoplasm PH, which would hinder the virus entry and replication in host cells.

Basically, CQ is the first 4-aminoquinolones reported to be effective in treating coronavirus infection *in vitro*. The infection of SARS-CoV-2 to target cells is mediated through the interaction of angiotensinconverting enzyme 2 (ACE2) and the viral spike protein (Al-Bari, 2015; Hoffmann et al., 2020). As shown in **Figure 3**, CQ could elevate the endosomal pH and interfere with terminal glycosylation of ACE2, which thus negatively affect the virus-receptor binding and abrogate the infection. When it comes to SARS-CoV-2, CQ could on the one hand block the interaction between virus “S” protein and host ACE2 protein through influence ACE2 terminal glycosylation and also increase the cytoplasmic PH, which thus inhibit the virus entry and replication (**Figure 3**) (Al-Bari, 2015; Clotilde et al., 2018; Hoffmann et al., 2020). CQ restriked public hope as the *in vitro* studies showed its EC50 for SARS-CoV-2 is 1.13 µM, half-cytotoxic concentration CC50 > 100 µM, with the selectivity index of 88.50, the result of which show high SI of CQ when treat COVID-19 and indicate desired safety (Wang et al., 2020). A preliminary clinical trial enrolled over 100 COVID-19 patients proved the efficacy and safety of CQ, and the seventh edition of Chinese Clinical Guidance for COVID-19 recommended the dosage of CQ as follows: 18–65 kg adults with a body weight of >50 kg, 500 mg orally twice daily, continuing for 7 days; body weight of <50 kg patients, taken 500 mg of CQ twice daily for the first 2 days and then followed by 500 mg once daily for the other 5 days (Cortegiani et al., 2020).

The structure of CQ and its analogs was shown in **Figure 2**. HCQ has the identical structure of CQ, while piperaquine (PQ) possesses the bisquinolines, which is trapped in the acidic digestive vacuole because of their four positive charges (Reuter et al., 2015). Here, we summerized the pharmacology parameters and concerns for these drugs and provide rational consideration for the clinical practitioners.

## Clinical Trials and Systematic Review of CQ and Its Analogs Against COVID-19

In fact, the activity of CQ against coronavirus has been noticed as early as 2004, as CQ has been proved to be effective to SARS-CoV *in vitro*, whose disappearance resulted in limited further research. In vitro research confirmed the efficacy of CQ against SARS-CoV-2 as well as remdesivir. The two compounds remdesivir (EC50, 0.77 µM; CC50 > 100 µM; SI > 129.87) and chloroquine (EC50, 1.13 µM; CC50 > 100 µM; SI > 88.50) potently blocked virus infection at low-micromolar concentration and showed high SI (Wang et al., 2020). Shortly afterward, Chinese researchers confirmed the antiviral activity of HCQ against SARS-CoV-2 and established [physiologically based pharmacokinetic (PBPK) models] PBPK models to optimize the dosage regimen. Xueting Yao et al. found HCQ (EC50 = 0.72 µM) was more potent than chloroquine (EC50 = 5.47 µM) *in vitro* and simulated PBPK model based on the above *in vitro* studies (Yao et al., 2020). According to their results, a loading dose of 400 mg twice daily of HCQ orally, followed by a maintenance dose of 200 mg twice daily for 4 days was recommended for COVID-19 patients, as it reached three times the potency of CQ 500 mg twice daily. Till now, there have been 14 clinical trials registered and approved in China to further investigate and compare the efficacy and safety of CQ or HCQ against COVID-19 (Al-Bari, 2015; Yao et al., 2020) (**Table 1**).

**Table 1:** Registered clinical trial of CQ and its analogs on treatment of COVID-2019.

No.	Registered no.	Title	Date of registration	Description for medicine or protocol of treatment in detail (number of enrolled subjects)	Primary sponsor
1	ChiCTR2000030718	Randomized controlled trial for Chloroquine Phosphate in the Treatment of novel coronavirus pneumonia (COVID-19)	2020/3/11	chloroquine phosphate(40); Control group(40)	Zhongnan Hospital of Wuhan University
2	ChiCTR2000030054	A prospective, open label, randomized, control trial for chloroquine or hydroxychloroquine in patients with mild and common novel coronavirus pulmonary (COVIP-19)	2020/2/22	Hydroxychloroquine sulfate 0.2g bid x 14 days a day(40): The first dose of chloroquine phosphate was 1gx2 days, and the third day was 0.5g x 12 days (40): Recommended treatment plan for novel coronavirus pneumonia diagnosis and treatment plan (20)	Zhongshan Hospital Affiliated to Xiamen University
3	ChiCTR2000029992	A prospective, randomized, open label, controlled trial for chloroquine and hydroxychloroquine in patients with severe novel coronavirus pneumonia (COVID-19)	2020/2/18	Hydroxychloroquine sulfate 0.2g bid x 14 days a day(40): The first dose of chloroquine phosphate was 1g x 2 days, and the third day was 0.5gx12 days (40): Recommended treatment plan for novel coronavirus pneumonia diagnosis and treatment plan (20)	Zhongshan Hospital Affiliated to Xiamen University
4	ChiCTR2000029988	Clinical Study of Chloroquine Phosphate in the Treatment of Severe Novel Coronavirus Pneumonia (COVID-19)	2020/2/18	Experimental group(40): Chloroquine phosphate; control group(40): No	Zhongnan Hospital of Wuhan University
5	ChiCTR2000029939	A Single-blind, Randomized, Controlled Clinical Trial for Chloroquine Phosphate in the treatment of Novel Coronavirus Pneumonia 2019 (COVID-19)	2020/2/16	Control group (50): The conventional treatment group will be treated according to the guidance of the “Diagnosis and Treatment Scheme of COVID-19” published by the National Health Commission; Intervention group (50): conventional treatment combined with chloroquine phosphate.	HwaMei Hospital, University of Chinese Academy of Sciences
6	ChiCTR2000029935	A Single-arm Clinical Trial for Chloroquine Phosphate in the treatment of Novel Coronavirus Pneumonia 2019 (COVID-19)	2020/2/16	Treated with conventional treatment combined with chloroquine phosphate (100)	HwaMei Hospital, University of Chinese Academy of Sciences
7	ChiCTR2000029899	Evaluation the Efficacy and Safety of Hydroxychloroquine Sulfate in Comparison with Phosphate Chloroquine in Mild and Commen Patients with Novel Coronavirus Pneumonia (COVID-19): a Randomized, Open-label, Parallel, Controlled Trial	2020/2/16	CQ: Day1: first dose: 6 tablets(0.1g/table); second dose: 6 tablets (0.1g/table)after 6 h; day 2–5: 2 tablets (0.1g/table), BID vs. HCQ: Days 1–3: 500 mg BID; days 4–5: 250 mg BID	Peking University Third Hospital
8	ChiCTR2000029898	Evaluation the Efficacy and Safety of Hydroxychloroquine Sulfate in Comparison with Phosphate Chloroquine in Severe Patients with Novel Coronavirus Pneumonia (COVID-19): a Randomized, Open-Label, Parallel, Controlled Trial	2020/2/16	Experiment group (50): Day 1: first dose: 6 tablets(0.1g/table); second dose: 6 tablets (0.1g/table)after 6 h; days 2–5: 2 tablets (0.1g/table); BID vs. control group (50): Days 1–3: 500 mg BID; days 4–5: 250 mg BID	Peking University Third Hospital
9	ChiCTR2000029868	Hydroxychloroquine treating novel coronavirus pneumonia (COVID-19): a multicenter, randomized controlled trial	2020/2/15	Treatment group(100): standard treatment according to the guideline recommendation combined with: Day 1 to day 3: oral hydroxychloroquine sulfate tablets (100mg/tablet), 4 tablets each time, 3 times a day; days 4 to 14: oral hydroxychloroquine sulfate tablets (100 mg/tablet), 4 tablets each time, 2 times a day. Control group (100): standard treatment according to the guideline recommendation.	Ruijin Hospital, Shanghai Jiaotong University School of Medicine
10	ChiCTR2000029803	A prospective, randomized, open-label, controlled clinical study to evaluate the preventive effect of hydroxychloroquine on close contacts after exposure to the Novel Coronavirus Pneumonia (COVID-19)	2020/2/14	Hydroxychloroquine, small dose (80); hydroxychloroquine, high dose (80);abidol hydrochloride, small dose (80); abidol hydrochloride, high dose (80)	Renmin Hospital of Wuhan University
11	ChiCTR2000029741	Efficacy of Chloroquine and Lopinavir/Ritonavir in mild/general novel coronavirus (CoVID-19) infections: a prospective, open-label, multicenter randomized controlled clinical study	2020/2/11	Chloroquine phosphate 56 vs. Lopinavir/Ritonavir 56	The Fifth Affiliated Hospital Sun Yat-Sen University
12	ChiCTR2000029609	A prospective, open-label, multiple-center study for the efficacy of chloroquine phosphate in patients with novel coronavirus pneumonia (COVID-19)	2020/2/6	1. Chloroquine phosphate tablet, 0.25 g per pill, which contains chloroquine 0.155 g; 2. Lopinavir/ritonavir tablet, each pill containing 200 mg of lopinavir and 100 mg of ritonavir mild-moderate chloroquine group 59 vs. mild-moderate Lopinavir/ritonavir group 59 vs. mild-moderate combination group 59; severe-chloroquine group14 vs. severe- Lopinavir/ritonavir group14	The Fifth Affiliated Hospital of Sun Yat-Sen University
13	ChiCTR2000029559	Therapeutic effect of hydroxychloroquine on novel coronavirus pneumonia (COVID-19)	2020/2/4	Hydroxychloroquine 0.1 oral 2/day;Hydroxychloroquine 0.2 oral 2/day vs. Starch pill oral 2/day	Renmin Hospital of Wuhan University
14	ChiCTR2000029542	Study for the efficacy of chloroquine in patients with novel coronavirus pneumonia (COVID-19)	2020/2/3	For the intervention group, oral chloroquine will be given on the basis of conventional management: the dosage is 0.5 g every time, twice a day for a 10-day course. For the control group, conventional treatment will be given.	Sun Yat sen Memorial Hospital of Sun Yat sen University

**Figure 4:**
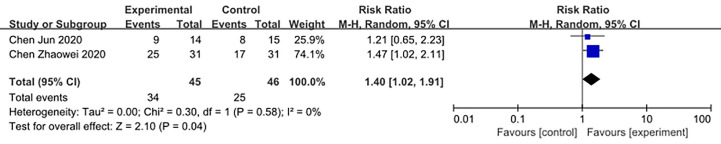
Forest plots of CT improvement.

**Figure 5:**
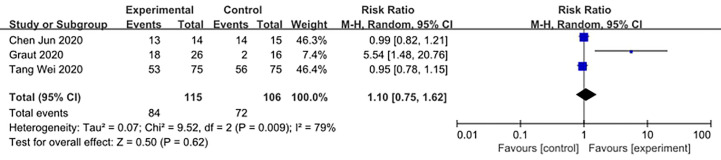
Forest plots of Virology negative conversion rate.

**Figure 6:**
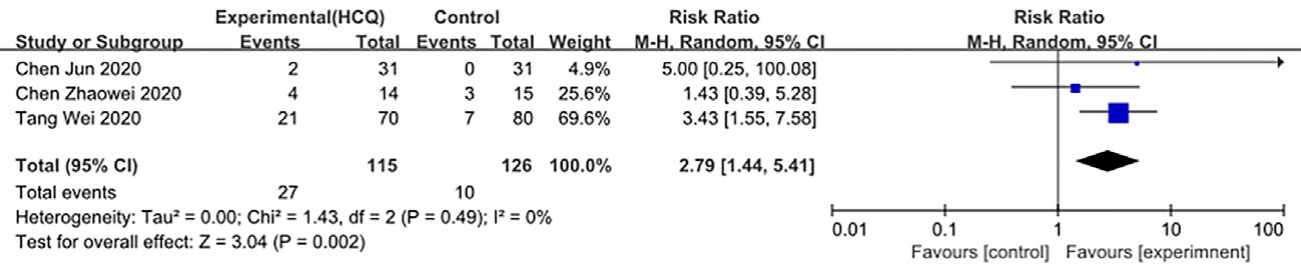
Forest plots of adverse reaction.

**Figure 7:**
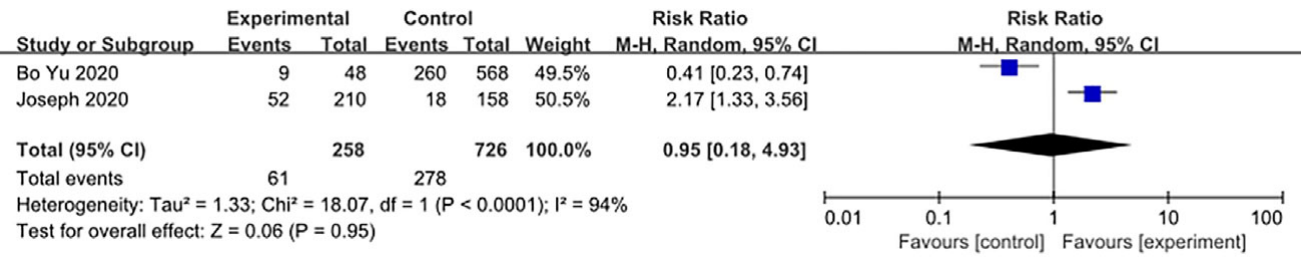
Forest plots of mortality.

We made database search and analyzed clinical study of HCQ treatment in COVID-19 worldwide and made a meta-analysis up to 4^th^ May, 2020. **Supplementary Figure 1** showed our study selection process. Finally, six papers included in our meta-analysis study. Characteristics of HCQ studies included in our meta-analysis were presented in **Table 2** (Chen et al., 2020a; Chen et al., 2020b; Tang et al., 2020; Gautret et al., 2020; Magagnoli et al., 2020; Yu et al., 2020). Meta-analysis was done by RevMan 5, and ramdom effect model was applied. As shown in **Figure 4**, CT scan indicated pulmonary improvement in 34/45 HCQ group, while there reported only 25/46 improvements in control group (P = 0.04). Additionally, there showed no significant differences in virology conversion rates when COVID-19 patients administered HCQ (P = 0.62) (**Figure 5**). Nevertheless, HCQ group had a higher risk ratio in inducing adverse reaction, which may due to the pharmacokinetic characteristics of HCQ (P = 0.002) (**Figure 6**). When it comes to mortality, the results of the two included study seemed to be opposite (**Figure 7**). Bo Yu et al. suggest that 400 mg of HCQ administered orally daily for 7–10 days could decrease the mortality, especially in critically ill patients, while the research work done by Joseph et al. did not tell the detailed regimen in experimental group. Since there was large heterogeneity in present clinical research, especially in virology conversion comparison and mortality, further high-quality, large-scale clinical study should be made to investigate the efficacy and safety in different subgroups. Above all, HCQ treatment could help alleviate pulmonary disease caused by COVID-19, and its higher incidence of ADRs should be warned in clinical applications. Wuhan Virology Institute warned the lethal dose of CQ was 3–5 g and 1g for adults and pediatrics, respectively (Bruno et al., 1988), and other side effects, such as liver and cardiac toxicity, is not uncommon among COVID-19 infected patients treated with CQ.

**Table 2:** Characteristics of studies included in the meta-analysis.

Study	Country	Design of the study	No. of patients (HCQ vs non-HCQ)	Median age	Treatment regimen in HCQ cohorts	Treatment regimen in non-HCQ cohorts
Chen J. et al. (2020)	China	Open, ramdomized	14/15	NA	400 mg qd for 5 days plus standard care	Standard care
Chen et al. (2020b)	China	Ramdomized	31/31	44.7(± 15.3)	400 mg qd for 5 days plus standard care	Standard care
Tang et al. (2020)	China	Open, ramdomized	70/80	46.1 ± 14.7	1200 mg loading for 3 days followed by a maintained dose of 800 mg for another 2–3 weeks	Standard of care
Yu et al. (2020)	China	Retrospective study	48/568	68(57–76)	400 mg (200 mg bid) for 7–10 days plus basic therapy	Basic therapy
Gautret et al. (2020)	France	Open, non-ramdomiazed	26/16	45.1(± 22)	600 mg of HCQ/d with or without azithromycin	Standard of care
Magagnoli et al. (2020)	United States	Retrospective study	210/158	70(59–75)	HCQ (dosage and duration unknown)	Standard of care

## Dosage Recommendations of CQ and Its Analogs

Basically, the approved uses of CQ is prophylaxis and treatment of malaria, non-intestinal amebic infection and sarcoidosis (Centers for Disease Control and Prevention (CDC), 2013; Product Information, 2017). 1000-mg initial dose was recommended and followed by 500 mg orally at 6, 24, and 48 h, the entire course of malaria treatment last no more than 2 days. For malaria prophylaxis, 500 mg orally once weekly was recommended 2 weeks prior to travel to the malarious area and continue for 4–8 weeks after leaving (Wang et al., 2020). When it comes to non-intestinal amebic infection and sarcoidosis, the dosage regimen and duration increased up to 3 weeks to 6 months, with maximum daily dosage of 500 mg. In the treatment of COVID-19, it is recommended to take 500 mg twice daily, continuing for 7 days for those weight above 50 kg for adults (18–65 years old) and for those weight below 50 kg for adults, it is recommended to take 500 mg twice daily for the first and second day and 500mg once daily for the next 5 days (2020).

HCQ has raised attention as it shares the identical parent structure of CQ and, most importantly, was reported to be less toxic. In the previous SARS outbreak, HCQ was reported to have anti-SARS-CoV activity *in vitro* as well (Coronavirus Task Force, 2020). The latest *in vitro* studies showed that HCQ is more potent for the treatment of COVID-19 than CQ (EC50 = 1.13 µM) as the EC50 of HCQ is 0.72 µM. PBPK modeling revealed that labeled dosage regimen (a loading dose of 400 mg twice daily of HCQ sulfate given orally, followed by a maintenance dose of 200 mg given twice daily for 4 days) could reach three times the potency of CQ phosphate when an HCQ sulfate of 500 mg is twice daily for 5 days in advance (Yao et al., 2020).

Though there is not a yet direct evidence for dihydroartemisinin-PQ (DHA/PQ) in antiviral therapy, we could predict it would have an identical activity as CQ, since PQ is the most active representative of bisquinolines, which used to overcome CQ resistance in malaria therapy (Kaur et al., 2010). Due to the four positive charges, PQ, which shares the bisquinolines structure, may be more efficiently trapped in the acidic digestive vacuole and thus inhibit coronavirus entry and replication (2020). Both population pharmacokinetic modeling and clinical practice demonstrate that DHA/PQ has better cardiac safety than CQ (Chotsiri et al., 2017; Vanachayangkul et al., 2017). Dihydroartemisinin also showed potent anti-virus activity (Gong et al., 2018). The dosage recommended for falciparum malaria treatment is based on weight, basically, 960 mg of PQ/120 mg of DHA orally once daily for 3 days for 36–75 kg adults (Product Information, 2012). Above all, DHA/PQ may serve as a rationale candidate in COVID-19 treatment.

## Pharmacokinetic of CQ andIts Analogs

Orally taking CQ is rapidly and almost completely absorbed from the gastrointestinal tract (Dattani and Rajput, 2010; Lee et al., 2012). Plasma protein binding of CQ is approximately 55%. After absorption, CQ is extensively distributed in body tissues and fluids. Besides, it could cross the placenta barrier and secrete into breast milk, which may be a risk for pregnancies and breast-feeding babies. It is mainly metabolized in the liver by CYP2C8, CYP3A4, and CYP2D6 with the metabolized product retained activity (Ducharme and Farinotti, 1996). The elimination half-life is as long as 108–291 h, with ~55% eliminated through the kidneys (Ducharme and Farinotti, 1996; Dattani and Rajput, 2010; Lee et al., 2012; Product Information, 2012). As HCQ has an identical structure of CQ, it possessed the same PK characteristics (high accumulation in cells and prolonged elimination half-life). Thus, a high loading dose and low maintenance dose was recommended to avoid adverse effects (Yao et al., 2020).

Additionally, there was also a clinical trial registered in China considering the application of DHA/PQ against COVID-19, though canceled due to lack of patients in the end (ChiCTR2000030082). Identical with CQ, DHA/PQ is mainly absorbed after taken orally, with peak plasma concentrations reached within 3–6 h for PQ and 1–3 h for DHA. PQ is then extensively distributed throughout the body, with a higher protein binding activity (more than 99% bound to plasma proteins). Besides, DHA has a smaller volume of distribution and plasma protein binding of 44–93%. The half-life of PQ was reported to be around (13.5–28days), while the elimination of DHA is much more rapid (with a half-life of ~1 h) (Kim et al., 2003; Hoglund et al., 2012).

Additionally, high-fat and high-calorie food intake could significantly increase the AUC and bioavailability of this drug, especially PQ, which may increase the risks of side effects. Consequently, it is recommended that DHA/PQ not be administered within ± 3 h of food consumption, especially for patients consuming a typical Western diet or taken with a low fat content of standard meals when co-administered with DHA/PQ (Tarning et al., 2008). The pharmacokinetic parameters of CQ and its analogs were shown in **Table 3**.

**Table 3:** Pharmacogenomics of CQ and its analogs (Roos et al., 2002; Li J. et al., 2020).

CQ and its analogs	Primary isoforms	Major metabolite
CQ	CYP2C8, CYP3A4/3A5, CYP2D6	Desethly derivative
HCQ	CYP2D6	Desethly derivative
Dihydroartemisin	UGT1A9, UGT2B7, CYP1A2	DHA-glucuronide
Piperaquine	CYP2E1, CYP3A4, CYP3A5, CYP2C19	Carboxyl acid cleavage product and mono-N-oxidated product

## Pharmacogenomics and Clinical Outcomes

With the development of genomics, the association of pharmacogenomics and anti-malarial drug metabolism have analyzed in recent years. CQ mainly metabolized by CYP3A4/CYP3A5 and CYP2C8 and CYP2D6, while only CYP2C8 SNPs were reported to influence clinical outcomes (Reuter, 2015). Additionally, CYP2D6 SNPs could influence the blood levels of HCQ (Elewa and Kyle, 2017). *In vitro* metabolism experiments revealed that CYP3A4 and CYP2C8 were the main metabolize enzyme of PQ, while CYP3A4 had a higher metabolic rate (Lee et al., 2016). Pharmacogenomic SNPs and pharmacokinetics of CQs as shown in **Table 4**. Additionally, CQ and HCQ were reported to be the substrate and inhibitor human organic anion transporting polypeptide 1A2, which believed to be one of the reasons for the vision impairment, as this effect may compromise the function of the classic visual cycle (Yao et al., 2020).

**Table 4:** PK parameters of CQ and its analogs.

Parameter	Drug			
	Chloroquine	Hydroxychloroquine	Piperaquine	Dihydroartemisinin
Bioavailability	89%	74.6%	~50%, fat food would increase the bioavailability	NA
Cmax (ng/mL)	283–1430	14(34.3 ± 9.5)	71.6–730	350(252–485)
Tmax (h)	2.7–6.9	4(3.65 ± 1.14)	1.48–5.7	3(2–60)
AUC (µg.h/mL)	8.2–140	1819 ± 417	24.1–49.5	
Elimination T1/2 (h)	108–291		13.5–28 days	332(174–631)
CL/F (L/h per kg)	0.23–0.8		0.85–1.85	
Vd/F (L/kg)	31.8–262		529–877	
Protein binding:	Approximately 55%	NA	Piperaquine is bound to human plasma proteins more than 99%, and dihydroartemisinin is 44 to 93% bound	
Renal excretion	One-fourth as desethylchloroquine, slightly more than half as unchanged drug (Chen et al., 2020a)	Renal: 16 to 62% unchanged	NA	NA
Day 7 concentration (ng/mL)			22.7–64	

## Safety

### Adverse Effects

CQ is generally well tolerated at therapeutic doses. Basically, liver function is the core element that guarantees the safety of all drugs (including CQ and its analogs). However, hepatotoxicity was not unusual in CQ treatment patients, even under the 10-days course treatment of COVID-19, which calls for careful monitoring liver function. That may due to the oxidative stress induced by CQ, which would elevate SDH, ATPase and ALKase activities (Yao et al., 2020) and thus lead to accumulation of CQ and induce various side effects.

Generally, the risk of side effects develops with the dosage of CQ accumulation *in vivo*. Pruritus is a common side-effect and is more severe in dark-skinned patients in previous anti-malarial and rheumatoid arthritis applications. Besides, headache, hepatitis, elevated liver enzyme, various skin eruptions, and gastrointestinal disturbances, such as nausea, vomiting, and diarrhea were also reported. Taking CQ with food helps to avoid gastrointestinal intolerance. The most severe side effects of CQ is cardiac toxicity, which was irreversible in some patients. Though more considerable doses accumulation was more frequently associated with cardiac side effects, patients with low dosage also reported having a quinidine-like effect arising from a severe valvular insufficiency (Li J. et al., 2020), and patients (especially aged patients) with abnormal liver function were more prone to induce cardiac toxicity mainly due to reduced CQ metabolism. The acute cardiac toxicity in high-dose administration or accumulation present as sodium, calcium, and potassium (hERG) channels blockade, which would lead to QT interval prolongation, widening QRS interval, and NO/histamine release (Clotilde et al., 2018). As pharmacogenomics involved mentioned before, potential drug-drug interactions, especially in COVID-19 treatment, should be avoided in clinical practice.

Additionally, some researchers seem to agree that CQ/HCQ-related cardiotoxicity was identical with Fabry disease (FD), as dysfunction of the lysosomal enzyme was also observed in CQ/HCQ-related cardiotoxicity (Roos et al., 2002; Putko et al., 2015). Roos et al. found clinicopathologic features of CQ/HCQ shared lots of similarity with FD, and the presence of curvilinear bodies was only found in CQ cardiotoxicity (Elewa and Kyle, 2017). Deficiencies of ɑ-Gal A were reported to be associated with Parkinson’s disease, which is a mental disorder that mainly occurs in the elderly (Nelson et al., 2018). These facts should be warned that elderly patients would be at a higher risk of side effects induced by CQ/HCQ, especially the fatal cardiac toxicity.

Besides, as renin-angiotensin system (RAS) inhibitors may elevate the expression of ACE2 in pulmonary, there is worrried about the disease of patients co-administered with RAS inhibitors aggravated. The research work done by Li et al. Demonstrate there was no association of severity or risk of deaths in COVID-19 patients with hypertension co-administered RAS inhibitors (Li J. et al., 2020). Actually, ACE2 was the homologue of ACE, inhibition of ACE would eventually increase the level of ACE2, which could augment the protective effect of vascular of heart, brain, kidney and pulmonary. Previous meta-analysis indicate ACE inhibitor could decrease risks of pulmonary and pulmonary induced death (Caldeira et al., 2012). In conclusion, currently it is not recommended COVID-19 patients with hypertension withdraw ACE inhibitors.

Retinopathy is another common side effect induced by CQ, with the incidence of HCQ lower than CQ (66.7% vs. 9.5%) (Clotilde et al., 2018). Retinopathy is irreversible side effects, and there is no present therapy, so it is of great importance to recognize at early stages and prevent central visual loss (Marmor et al., 2016). Asian patients were reported to show an extravehicular pattern of damage while the locus of toxic damage is paralegal in many eyes as well (Marmor et al., 2016).

Nguyen et al. pointed out if CQ is over 2.3 mg∙kg^-^1 daily dosage[g] or HCQ is over 5 mg∙kg^-^1 daily dose (Marmor et al., 2016), the incidence of retinopathy induced by CQ would increase. Besides high doses and long duration, renal disease or use of tamoxifen, which is metabolized by CYP2D6, were another two major risk factors of retinopathy (Marmor et al., 2016; Nguyen et al., 2018). CYP2D6 is the main metabolizer of CQ/HCQ, and tamoxifen may induce competitive antagonist of CQ/HCQ, which consequently lead to accumulation of these drugs *in vivo*.

As regimens of large dosages and short durations CQ/HCQ was registered in some clinical trials, the retinopathy should be carefully monitored, and CQ should not be used in patients with histories of retinopathy or vision decline.

**Figure 8:**
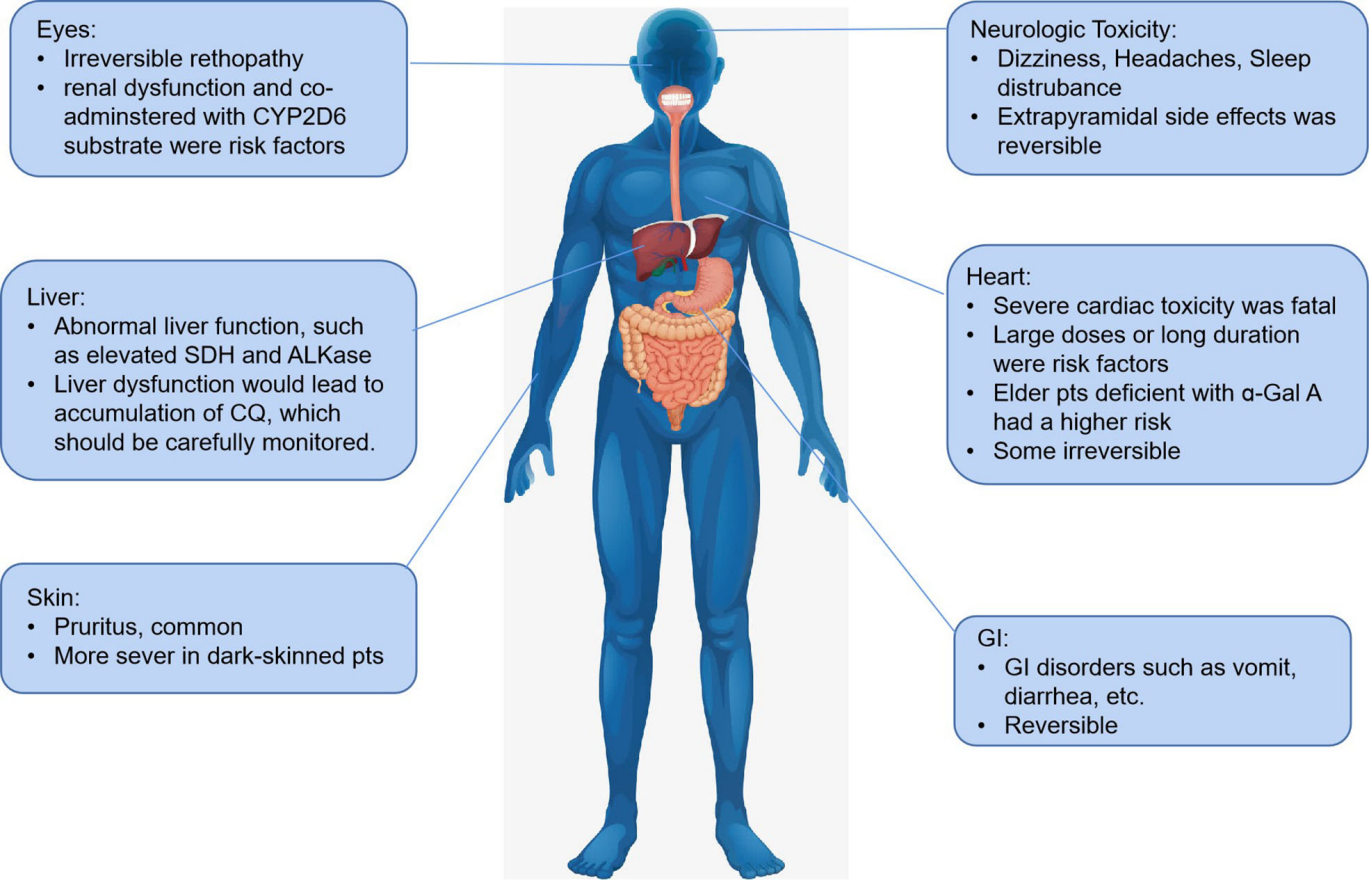
Adverse effects induced by CQ/HCQ.

Neurologic toxicities including primarily headache, dizziness, and sleep disturbance were reported. Though central nervous system toxicity such as convulsions and mental changes occurred more rarely, it should not to be neglected. Systematic review done by Sandipan suggests that CQ may not increase the risk of seizure (Pati, 2020). These extrapyramidal side effects were reversible as the symptom disappearred after the drug withdrawal. Adverse reactions induced by CQ/HCQ were shown in **Figure 8**.

When it comes to HCQ, basically HCQ is less toxic to CQ, especially the retinopathy incidence. Higher or long treatment (e.g., treatment duration longer than 5 years for anti-malarial treatment) could emerge side effects. A systematic review reported HCQ could cause cardiac disorder, especially in high doses or accumulated high doses, which may provoke potentially irreversible damage and death (Xu et al., 2016). Ingestion of 8–22 g by adults has caused life-threatening toxicity (e.g., dysrhythmias, hypotension, and coma). Considering the long elimation half-life and cumulative effect, side effects should be monitored carefully.

Generally, DHA/PQ was reported to be well tolerated in previous research, though adverse drug reaction such as gastrointestinal complaints (nausea, diarrhoea, and vomiting, as well as anorexia) and nervous system side effects (dizziness, headache, sleep disturbance, etc) was not uncommon (Yam and Kwok, 2006; LiverTox, 2012). Similar with CQ, in anti-malaria treatment, DHA/PQ could cause QT interval prolongation in some patients at the labeled doses, though no torsade de pointes (TdP) were noticed (Yavo et al., 2011). Besides, high fat food intake would significantly elevate the bioavailability of DHA/PQ, which may increase the risks of cardiac toxicity (Li et al., 2019). Consequently, it is recommended to take low-calorie food during DHA/PQ therapy, or DHA/PQ should not be administered within ± 3 h of food consumption (Yavo et al., 2011). There is no report of lethal dose of DHA/PQ. Rhesus monkey *in vivo* experiments demonstrated the no-observed-adverse-effect level for DHA/PQ was considered to be 39.1 mg/kg (Li et al., 2019).

In conclusion, as CQ and its analogs were the inhibitors or substrate of various CYP450 enzymes and could cause lethal cardiac toxicity in large accumulated doses, it is recommended not to co-administered drugs that may have cardiac side effects (such as moxifloxacin) with CQ and its analogs. Demonstrated drug interactions were shown in **Table 5**. Additionally, if the treatment courses of CQ to COVID-19 are longer than malaria, then the potential side effects should be monitored carefully so as to avoid adverse drug interactions. Besides, as CQ and its analogs could inhibit the activity of CYP3A4/3A5, the blood concentration of immunosuppressants, such as tacrolimus and cyclosporin, should be monitored more frequently in COVID-19 patients with solid organ transplantation history. Last but most important, as liver damage occurred especially in critical severe COVID-2019 infected patients (Li et al., 2019), the metabolism of CQ and its analogs would be further reduced and then increase the accumulation of those drugs *in vivo*, which may in turn lead to a higher incidence of various side effects and should be warned by clinicians.

**Table 5:** Demonstrated interactions of CQ and its analogs (Li J. et al., 2020).

Antimalarial drug	Demonstrated interactions	Potential interactions
Chloroquine	Increased plasma concentration with paracetamol Reduced metabolism and clearance with cimetidine Reduced absorption with antacids and kaolin Reduced bioavailability of ampicillin and praziquantel; Reduced therapeutic effect of thyroxine; Increased plasma concentration of cyclosporine; Reduced plasma concentration of methotrexate; Reduced antibody response to primary immunization with intradermal human diploid-cell rabies vaccine	Increased convulsions with mefloquine; Increased risk for acute dystonic reactions with metronidazole; Antagonises effects of antiepileptics
Hydroxychloroquine	Increase risk of QT-interval prolongation; QT interval prolonging drugs (mesoridazine\thioridazine); Increase risk of torsades de pointes: amisulpride Increase risk of blood dyscrasias: aurothioglucose Increase serum digoxin concentrations: Digoxin Reduce bioavailability of antimalarials: Lanthanum carbonate	
Dihydroartemisinin	Reduced plasma concentration when artesunate or artemether is given with ritonavir	May result in slight decrease in CYP1A2 activity
Piperaquine	Piperaquine thtraphosphate/dihydroartemisinin: Increase exposure of piperaquine and CYP3A4 substrates: CYP3A4 inhibitors (abiraterone acetate\alprazolam) Decreased exposure of piperaquine: CYP3A4 inducer (amprenavir\armodafinil) Decrease exposure of CYP2E1 substrates: CYP2E1 inducer(acetaminophen\chlorzoxazone) Increase exposure of CYP2C19 substrates: CYP2C19 inhibitor(amitriptyline\carisoprodol) Increase risk of QT-interval prolongation: QT interval prolonging drugs(alfuzosin\anagrelide) Increase exposure of CYP1A2 substrate: aminophylline Increase risk of fatal arhythmias: amisulpride	Increased plasma concentration and potential increased toxicity with verapamil, indinavir, lopinavir + ritonavir, HMG-CoA reductase inhibitors (statins) and cyclosporina In-vitro metabolism by CYP3A4 increased (60) and thus reduced plasma concentration and potentially reduced effectiveness with barbiturates, chronic alcohol use, rifampicin, efavirenz, nevirapine, phenytoin or carbamazepinea Increased risk for cardiac events with drugs that prolong the QTc intervala

## Dosage Considerations for Other Patients

### Elderly

Chineses scientists have dedicated themselves to rescuing COVID-19 patients, and Li Xiaochen et al. found age above 45, hypertension, high lactose dehydrogenase, and D-dimer level were risk factors for severity and mortality in adult COVID-19 patients (Li X. et al., 2020). Actually, the National Health Commission of China has notified more than 80% of the deaths are older people over 60 years old, and more than 75% of the deaths have underlying diseases (Rico-Mesa et al., 2020). Clinical studies have also confirmed that elderly patients with comorbidities such as hypertension, cardiovascular diseases, diabetes, chronic respiratory disease and chronic kidney disease (CKD) had a striking case fatality rate (CFR)[d], which rises up to 13.2% for patients with cardiovascular disease especially (WHO-China Joint Mission; Shahid et al., 2020).

Angiotensin-converting enzyme inhibitors (ACEI) and angiotensin II receptor blockers (ARB) could upregulate the expression of the ACE-2 receptor, which was the critical receptor that the SARS-CoV-2 virus utilized to enter human cells (Lo et al., 2020). Though scientists worried about ACEI and ARB may play a negative role in COVID-19 treatment based on *in vitro* and *in vivo* results[c], further clinical observations did not find ARB and ACEI could increase CFR (Rico-Mesa et al., 2020; Zhang et al., 2020). Consequently, significant cardiology scientific associations, including ACC, HFSA, AHA, and ESC Hypertension Council, not advised to stop ACEI/ARB administration in COVID-19 patients. However, since CQ and HCQ had cardiovascular toxicity, elderly patients above 60 years old, especially with cardiovascular system comorbidities, should be monitored carefully. It was recommended in expert consensus on novel coronavirus pneumonia in the elderly that released by National Geriatric Medical Center in China, drug-interactions and pharmacokinetics should be concerned carefully when administered antiviral agents and CQ/HCQ in elderly patients (2020).

### Pediatrics

According to epidemiological research of COVID-19 among pediatrics in China, children of all ages were susceptible to COVID-19, though clinical manifestations were generally less severe, young children, particularly infants, were more vulnerable to SARS-CoV-2 infection (Dong et al., 2020). As CQ constitute the clinical treatment of COVID-19 in many countries, pediatrics dosing guidelines were urgently needed. As a traditional antimalaria agent, WHO has released guidelines for the treatment of malaria; however, there was little content concerning the efficacy and safety of CQ treatment in pediatrics. Laurens et al. reported CQ dosing recommendation for pediatrics based on PBPK modeling: 35 mg/kg (CHQ base) for children 0 to 1 month, 47 mg/kg for 1–6 months, 55 mg/kg for 6 months–12 years, and 44 mg/kg for adolescents and adults, not to exceed 3,300 mg in any patient (Verscheijden et al., 2020). With cautions that the lethal toxicity in children has been reported as unintentional overdoses of ~35–100 mg/kg of CQ (base) in a single dose (Verscheijden et al., 2020). Currently, diagnosis and treatment recommendation for pediatric COVID-19 released by China National Clinical Research Center for Child Health was not recommended CQ in pediatrics. Besides, pediatircs were listed as contraindication population in expert consensus on chloroquine phosphate for the treatment of novel coronavirus pneumonia (2020). Above all, pediatrics were generally not recommended to be administered CQ due to its toxicity and narrow therapeutic window. If CQ was needed in urgent circumstances, then the side effects of CQ should be monitored carefully.

### Pregnancy

CQ and other 4-aminoquinolones could cross the placenta. Although there is reported that at the labeled doses (Caldeira et al., 2012; Marmor et al., 2016), the fetal risk could be ruled out. The potential benefits of drug treatment against potential risks should be weighed before prescribing this drug to pregnancies, and the response and toxicity should be monitored more closely (European Eedicines Agency, 2019; Fan et al., 2020). As CQ is toxic to the myocardium and could be accumulated in the melanin of the fetus eyes, it is cautious to administered CQ to pregnant women due to its toxicity in fetal formation and other irreversible damage.

As malformations were not reported in animals administered PQ, PQ may be safe in reasonable dosage ranges. However, delay of delivery, which may induce neonatal mortality, was reported during peri- and postnatal development studies with 80 mg/kg of PQ. The safety of DHA is controversial, as embryo lethality and teratogenicity have been reported in animals administered DHA, although recent systematic review showed the effect of DHA/PQ (especially monthly) on low birth weight and adverse birth outcomes was minimal (Xu et al., 2016; Pati, 2020). In this case, it is recommended do not administer DHA/PQ combination to a pregnant woman if other anti-malaria treatments are available through animal studies revealed that male and female fertility are unaffected by the presence of DHA (Olaleye et al., 2019).

### Breast-Feeding

CQ and HCQ have been found in low amounts in breast milk, and several studies have reported no adverse effects of this drug in infants exposed during the lactation period. Although the benefits of breastfeeding outweigh the theoretical risk to the infant, the nursing infant should always be monitored for adverse effects (Massele et al., 1997; Anon, 2002; Pati, 2020). Animal studies suggest that PQ may be excreted into breast milk, though it is unknown whether DHA/PQ combination is excreted into human breast milk. It should be carefully weighed the potential benefits of drug treatment against potential risks before prescribing this drug during breast-feeding (Xu et al., 2016).

## Conclusion

Since COVID-19 is warned by WHO as a pandemic around the world, it is of great importance to explore active drugs. CQ and its several analogs, especially HCQ, showed promising antiviral activity *in vitro* and preliminary clinical studies confirmed its efficacy. Nevertheless, adverse effects induced by CQ or its analogs should not be ignored due to their long half-lives and are lethal in large accumulated doses. As CQ or its analogs were inhibitors or substrate of CYP450, we suggest clinicians to assess the drug-to-drug interactions before use so as to avoid severe side effects, such as cardiac toxicity, liver damage, ocular toxicity, etc. Though CQ or HCQ had not shown significant advantages in virology conversion, the immuno-regulatory effect may help the body to combat the COVID-19. When it comes to the special population, such as the elderly, pregnant women, it should be carefully weighed the potential benefits of drug treatment against potential risks before prescribing. The metabolism of the elderly and the pediatric patients varied from the adults, which should be taken into full consideration. Additionally, patients with cardiac disease should be ruled out and monitored the side effects more carefully.

Recently, the US FDA has revoked the emergency use authorization (EUA) that allowed for CQ and HCQ to treat certain hospitalized patients with COVID-19 due to the emerging scientific data. The US FDA announced that “In light of ongoing serious cardiac adverse events and other potential serious side effects, the known and potential benefits of CQ/HCQ no longer outweigh the known and potential risks for the authorised use;” however, the efficacy and safety of CQ/HCQ in COVID-19 is controversial. The Lancet journal has retracted a large study of HCQ after finding flaws in the dataset, which published on 22 May. The study found the drug did not help treat COVID-19. Chinese scientists Nanshan et al. reported their study compared the efficacy of CQ in COVID-19 treatment (Huang et al., 2020). They enrolled 197 patients completed CQ treatment, and 176 patients were included as historical controls. The median time to achieve an undetectable viral RNA was 6-day shorter in chloroquine than in non-chloroquine. No serious adverse events were observed in the chloroquine group.

Above all, high-quality, large-scale clinical research still need to be done to assess the potential benefits and risks for different patients, and PBPK modeling may help optimize the clinical dosing design. It is of great importance for all the clinical participants or coordinates together to overcome the COVID-19 crisis.

## Author Contributions

HP, ZC, SR, JW, MZ, CZ, LD, YZ, and LC prepared the manuscript and do the literature research. HP, ZC, TX, and XL wrote the manuscript. JH, XW, and TH did the article review and the corresponding author TH and XW approved the final version. All authors contributed to the article and approved the submitted version.

## Conflict of Interest

The authors declare that the research was conducted in the absence of any commercial or financial relationships that could be construed as a potential conflict of interest.
