# Supplementary material for: Systematic Review and Pharmacological Considerations for Chloroquine and Its Analogs in the Treatment for COVID-19
Source: Front Pharmacol. 2020 Oct 28;11:554172. doi: 10.3389/fphar.2020.554172 (PMC7655531; doi:10.3389/fphar.2020.554172)
Supplement: Supplementary file 1 [file Image_1.pdf]

Supplementary Figure Legend

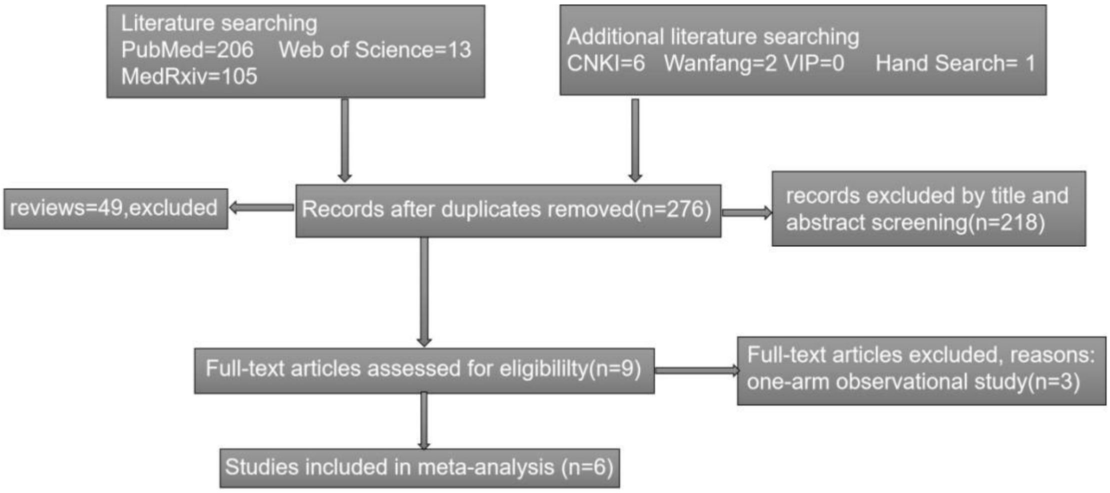

Supple Figure 1 PRISMA flow diagram for the study selection process
